# Extraordinary Creatine Phosphokinase Levels in Coxsackie B Necrotizing Myopathy Complicated by Rhabdomyolysis

**DOI:** 10.7759/cureus.25201

**Published:** 2022-05-22

**Authors:** Jacob Alex, Elise Landa, Arjun Trivedi, Leo M Parsons II, Nabeel Shabo

**Affiliations:** 1 Internal Medicine, Ascension Providence Hospital, Southfield, USA; 2 Critical Care Medicine, Ascension Providence Hospital, Southfield, USA; 3 Infectious Diseases, Ascension Providence Hospital, Southfield, USA

**Keywords:** creatine phosphokinase, acute renal failure, necrotizing myopathy, rhabdomyolysis, coxsackie b

## Abstract

Coxsackie B infections can have varying clinical presentations. Necrotizing myopathy and rhabdomyolysis with remarkably high creatine phosphokinase levels is a rare complication associated with high morbidity and mortality. A 28-year-old male presented with complaints of weakness, body aches, and decreased urine output. Initial lab work showed a creatine phosphokinase level estimated at 5,366,100 U/l. Initial Coxsackie B4 titers were at 1:160. Muscle biopsy of the right calf revealed necrotizing myopathy consistent with viral myopathy. This case highlights Coxsackie B4 as a potential pathogen that can cause extensive muscle necrosis producing extreme creatine phosphokinase levels leading to rhabdomyolysis. Taking a comprehensive history is essential to identify viral prodromal symptoms to guide broader serological testing for uncommon viral species.

## Introduction

Viral infections are an uncommon but well-documented cause of rhabdomyolysis, of which influenza types A and B are the most common culprits [1]. Coxsackie virus-induced rhabdomyolysis is a rare phenomenon. Coxsackieviruses are classified as RNA enteroviruses belonging to the Picornaviridae family. In the adult population, it typically produces a nonspecific, often asymptomatic infection characterized by low-grade fever [2]. In this case report, we present a previously healthy young man who developed a Coxsackie B viral infection leading to rhabdomyolysis and acute renal failure with a presenting creatinine kinase level of over five million U/l.

## Case presentation

The patient was a 28-year-old male with no significant past medical history who presented with complaints of left lower leg pain and severe weakness. He had also experienced oliguria and dark urine for the same period of time. For three days, he had been unable to walk and needed his brother to carry him to the bathroom. Two weeks prior to these symptoms, he had experienced malaise, fevers, and rhinorrhea. The patient did not report any family history of myopathy. He was not on any home medications and he denied any history of recreational drug use or strenuous exercise. On presentation to the emergency department, his vitals were temperature of 99.4°F, heart rate of 98 beats per minute, blood pressure 144/66 mmHg, respiratory rate of 20 breaths per minute, and oxygen saturation of 95% on room air. The patient was only oriented to self and had clouding of consciousness. Physical exam was notable for strength of 2/5 in bilateral lower extremities and diffuse tenderness in the lower extremities. Initial lab work was notable for blood urea nitrogen (BUN)/creatinine 36/4.4 mg/dL, lactate dehydrogenase (LDH) 13,120 U/l, lactic acid 2 mmol/L, and an initial creatinine kinase level of 5,366,100 U/l, and serum potassium level of 6.5 mmol/L. He was in acute renal failure secondary to rhabdomyolysis with severe metabolic derangements requiring emergent hemodialysis.

**Figure 1:**
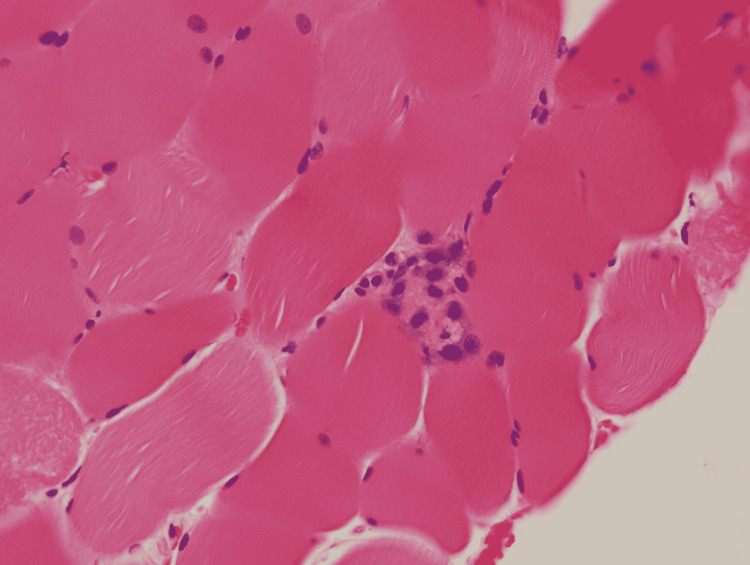
Right calf muscle biopsy showing necrotizing myopathy

Infectious workup including the following, urine culture, blood culture, and cerebrospinal fluid culture showed no growth. Lumbar puncture was done, and analysis of cerebrospinal fluid was unremarkable. Respiratory viral panel polymerase chain reaction (PCR) negative, viral hepatitis panel, Legionella antigen, and QuantiFERON negative. Autoimmune workup: antinuclear antibody (ANA), c-antineutrophil cytoplasmic autoantibodies (ANCA), p-ANCA, cardiolipin, double-stranded DNA (dsDNA), mitochondrial antibody, Sjogren's syndrome A (SSA)/Sjogren's syndrome B (SSB) antibody, smooth muscle antibody, complement C3, and C4 levels were all within normal range. Additional testing for unusual viral pathogens resulted in a positive IgG titer of 1:180 for Coxsackie B type 4. Serological testing for hepatitis C, Epstein-Barr virus, and cytomegalovirus were negative. Muscle biopsy of right calf showed pathological evidence of necrosis of skeletal muscle tissue (Figure 1). Over the 20-day hospitalization, he continued to have dialysis and supportive treatment for the viral illness with some resolution of symptoms. He remained on dialysis and was transferred to a long-term acute care facility for continued care. 

**Figure 2:**
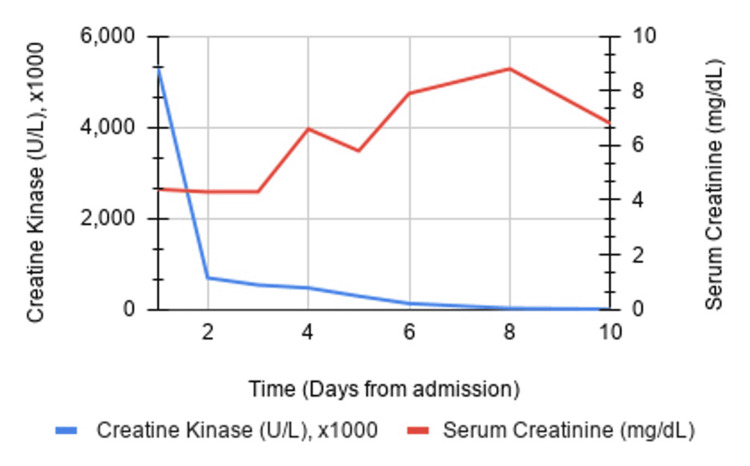
Creatine kinase and serum creatinine level trends during initial 10 days of inpatient admission

 After one hemodialysis session, creatinine kinase levels had decreased to approximately 500,000 U/L (Figure 2). 

## Discussion

Rhabdomyolysis is an uncommon manifestation of Coxsackie B viral infection. Injury to skeletal muscle releases intracellular contents into the bloodstream, including creatinine kinase, myoglobin, phosphate, and potassium. Myoglobin reaches the kidneys, and as water is reabsorbed in the tubules, myoglobin forms a precipitate leading to tubular obstruction and renal damage [3]. 

What is peculiar about this case is the exceptionally high presenting creatinine kinase level of 5,366,100 U/L. A case series by Melli et al., evaluating 475 patients with rhabdomyolysis from varying etiologies, found peak creatinine kinase levels ranging from 10,000 to 25,000 U/L [4]. Our literature review indicated the highest recorded level of creatinine kinase was 701,400 U/L in a case of Legionella pneumophilia pneumonia sepsis and acute renal failure [5]. In our patient, we suspect the inciting event was the Coxsackie B4 infection, as indicated by the patient having prodromal symptoms two weeks prior to deteriorating at home and confirmed by positive IgM. Typical complications of Coxsackie B infections are aseptic meningitis, myopericarditis, and pancreatitis [6-8]. Coxsackie B4 is associated with higher mortality compared to other serotypes [9]. Muscle biopsy showed pathological evidence of necrotizing myopathy, which is an uncommon complication of Coxsackie B4. We suspect high creatinine kinase levels were primarily due to massive necrosis of skeletal muscle, coupled with extended time bedbound with poor oral intake and worsening oliguria. The lack of early treatment accelerated the buildup of creatinine kinase in the bloodstream and worsened kidney injury. After one hemodialysis session, creatinine kinase levels had decreased to approximately 500,000 U/L.

A second explanation for exceedingly high creatinine kinase levels is direct damage of renal tissue by the Coxsackie B virus. The receptor which coxsackie B virus binds to on eukaryotic cells and viral RNA polymerases have been identified in renal tissue [10]. Furthermore, it has been shown that the specific serotype coxsackie B4, when injected into mice, produces mesangial proliferative glomerulonephritis [11]. Concomitant infection of both skeletal muscle and renal tissue could explain high creatinine kinase levels and acute renal failure requiring extended hemodialysis. Only a handful of case reports are present describing coxsackie B virus renal disease; more investigation is needed.

## Conclusions

As seen in this case, with accurate history taking, we were able to determine that the patient had a preceding viral illness. This information is what guided further investigations into unusual pathogens. Oftentimes when we are faced with patients in critical conditions, we reflexively treat the physical and metabolic abnormalities we find. It is essential to take a comprehensive history to identify viral prodromal symptoms, which could necessitate broader serological testing for unusual viral species.
